# Key points in pulmonary metastasectomy from colorectal carcinoma: European Society of Thoracic Surgeons survey analysis

**DOI:** 10.1093/icvts/ivad017

**Published:** 2023-01-23

**Authors:** Giuseppe Cardillo, Sara Ricciardi, René Horsleben Petersen, Richard Stephen Milton

**Affiliations:** Unit of Thoracic Surgery, San Camillo Forlanini Hospital, Rome, Italy; Unicamillus–Saint Camillus University of Health Sciences, Rome, Italy; Unit of Thoracic Surgery, San Camillo Forlanini Hospital, Rome, Italy; Alma Mater Studiorum, Univeristy of Bologna, Bologna, Italy; Department of Cardiothoracic Surgery, Copenhagen University Hospital, Rigshospitalet, Copenhagen, Denmark; Department of Thoracic Surgery, St James's University Hospital, Leeds, UK

##  

van Dorp and all co-authors of the survey on metastasectomy for colorectal (CRC) pulmonary metastases among members of the European Society of Thoracic Surgeons (ESTS) should be commended for an excellent paper with very interesting take-home messages for the whole thoracic surgery community.

Surgical resection is a common curative-intent procedure for selected patients with lung metastases from CRC with a controversial survival benefit [1, 2].

In their survey, containing 38 questions with 321 responses (with a 22.4% response rate), the authors highlight the evolution of the surgical attitude in the treatment of colorectal metastases when comparing the present examination to a previous ESTS survey, which dates back to 2008 and evaluated 146 respondents (with a 29.6% response rate) [3].

The main difference between the 2 ESTS surveys is the change in practice from open to minimally invasive approach: in the current survey, only 27.6% of the surgeons preferred a bimanual open palpation of the lung versus 65% of the surgeons in 2008 ESTS survey. The widespread adoption of a minimally invasive approach is the first key point presented in this survey: 88% and 81% of the surgeons use VATS/RATS respectively in unilateral and bilateral lesions. This figure should also be compared with the rate of 37% minimally invasive technique from the ESTS 2007–2019 database [4] and with the 74% reported from the Dutch Lung cancer Audit evaluating the 2012–2019 timeframe [5].

The attitude favouring minimally invasive approach showed in the present study is mainly due to the extensive use of this approach in thoracic surgery and to the refinements of new CT scanners, which have highly enhanced the precise anatomic knowledge and the location of lung nodules. Further improvement will be provided by 3D imaging technology [6]. An additional input to VATS/RATS has been prompted by the uptake of anatomic segmentectomy, which has a role not only in the centrally located nodule but also in most of the lesions with unknown preoperative or intraoperative clear diagnosis. We know from the present ESTS survey that tissue biopsy is rarely or never performed preoperatively, according to 68.6% of participants. It is also clear that intraoperative pathological examination may not differentiate between primary or secondary cancer, so that anatomic segmentectomy appears to be the optimal approach for metastases as well as for primary lung neoplasm [7].

A second key point to be stressed is the need for lymph nodal evaluation before and during surgery. Preoperative mediastinal assessment is done with EBUS/mediastinoscopy in 46% of the cases with N1 lesions and in 71% of the cases with N2 lesions. During surgery, 67.1% of surgeons perform lymph node assessment (sampling/dissection). More surgeons are inclined to perform lymph node assessment for central metastases compared to peripheral metastases (79.3% vs 56.4%, *P* < 0.001).

Probably the most important take-home message from the survey is the importance of multidisciplinary tumour board (MDT) discussion: a very high percentage (90%) of respondents regularly review pulmonary metastasectomy cases in an MDT. Tumor board meetings provide prospective patient case review and assure quality of care, evaluation related to diagnosis, treatment, symptom management, follow-up, prehabilitation and/or rehabilitation, and supportive care. Discussion includes expert clinical opinions with correct diagnostic work-up, treatment recommendations and opportunities for clinical trial participation.

Concerning diagnostic work-up, 81.5% of participants always or usually perform preoperative PET–CT and this represents an impressive number revealing a high level of care and the presence of oncologists in the set-up.

Furthermore, the need for MDT discussion is raised by the new trends in immunotherapy and by the doubts regarding the clear survival benefit related to pulmonary metastasectomy according to recent data. For most surgeons (91.6% of the respondents in the ESTS survey), survival represents the main reason for surgery in CRC metastases; however, the benefits of metastasectomy have been questioned by the Pulmonary Metastasectomy in Colorectal Cancer (PulMiCC) trial and by a recent systematic review [1, 2]. PulMiCC is a randomized, controlled noninferiority trial, which showed in 93 patients from 2010 to 2016 a 5-year survival of 47% after pulmonary metastasectomy vs 22% for the non-surgical group. This study, although deemed underpowered to test non-inferiority, should at least be taken into account for multidisciplinary decision-making, because it has cast doubt in the assumption of zero survival without metastasectomy. Patients in the surgical group showed favourable prognostic factors (ECOG zero; better FEV1; CEA < 5 ng/ml; no liver metastases) that represent confounding factors in the evaluation of the long-term results [2]. Furthermore, a very recent meta-analysis of survival outcomes following surgical and non-surgical treatments for pulmonary CRC metastasis on 2232 patients showed comparable survival among the 2 groups [1]. Significant selection bias contributes to this finding, prompting the need for high-powered randomized controlled trials and large registry data.

Concerning immunotherapy, a recent systematic review and meta-analysis on clinical benefits of PD-1/PD-L1 inhibitors in patients with metastatic CRC show that immunotherapy inhibitors have positive long-term outcomes effects: this finding can widely change the therapeutic situation of metastatic colorectal carcinoma [8].

A declared limitation of this survey—which is a common finding of all surveys—is the nonresponse bias: surgeons who perform metastasectomy are more likely to participate.

A second flaw, according to our opinion, is the absence of questions on safe resection margins: the risk of resection margin recurrence appears to be an important concern for wedge resections. Recent studies show that a narrow margin is a prognosticator for both survival and recurrence [9, 10].

In conclusion, the current ESTS survey displays a contemporary approach of European surgeons in the treatment of patients affected by pulmonary metastases from CRC (Supplementary Material).

Perhaps future surveys could involve the wider multidisciplinary team, reflecting the involvement of oncologists as well as surgeons in the treatment of pulmonary metastases from colorectal carcinoma.

## Supplementary Material

ivad017_Supplementary_Data
