# Supplementary figures and images for: Key points in pulmonary metastasectomy from colorectal carcinoma: European Society of Thoracic Surgeons survey analysis
Source: Interdiscip Cardiovasc Thorac Surg. 2023 Jan 23;36(2):ivad017. doi: 10.1093/icvts/ivad017 (PMC9932001; doi:10.1093/icvts/ivad017)

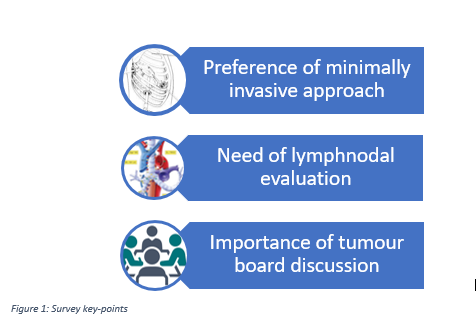

Supplement: ivad017_Supplementary_Data [file ivad017_supplementary_data.zip › ICVTS_imm.png]
